# Natural variations of heterosis-related allele-specific expression genes in promoter regions lead to allele-specific expression in maize

**DOI:** 10.1186/s12864-024-10395-y

**Published:** 2024-05-14

**Authors:** Weimin Zhan, Lianhua Cui, Shuling Yang, Kangni Zhang, Yanpei Zhang, Jianping Yang

**Affiliations:** 1 College of Agronomy, Henan Agricultural University, Zhengzhou, 450002 China; 2 Guangdong Provincial Key Laboratory of Plant Adaptation and Molecular Design, Guangzhou Key Laboratory of Crop Gene Editing, Innovative Center of Molecular Genetics and Evolution, School of Life Sciences, Guangzhou University, Guangzhou, 510006 China

**Keywords:** Maize, Heterosis, Allele-specific expression, Promoter variation, Domestication

## Abstract

**Background:**

Heterosis has successfully enhanced maize productivity and quality. Although significant progress has been made in delineating the genetic basis of heterosis, the molecular mechanisms underlying its genetic components remain less explored. Allele-specific expression (ASE), the imbalanced expression between two parental alleles in hybrids, is increasingly being recognized as a factor contributing to heterosis. ASE is a complex process regulated by both epigenetic and genetic variations in response to developmental and environmental conditions.

**Results:**

In this study, we explored the differential characteristics of ASE by analyzing the transcriptome data of two maize hybrids and their parents under four light conditions. On the basis of allele expression patterns in different hybrids under various conditions, ASE genes were divided into three categories: bias-consistent genes involved in basal metabolic processes in a functionally complementary manner, bias-reversal genes adapting to the light environment, and bias-specific genes maintaining cell homeostasis. We observed that 758 ASE genes (ASEGs) were significantly overlapped with heterosis quantitative trait loci (QTLs), and high-frequency variations in the promoter regions of heterosis-related ASEGs were identified between parents. In addition, 10 heterosis-related ASEGs participating in yield heterosis were selected during domestication.

**Conclusions:**

The comprehensive analysis of ASEGs offers a distinctive perspective on how light quality influences gene expression patterns and gene-environment interactions, with implications for the identification of heterosis-related ASEGs to enhance maize yield.

**Supplementary Information:**

The online version contains supplementary material available at 10.1186/s12864-024-10395-y.

## Background

Hybrid vigor, or heterosis, is a phenomenon where hybrid offspring exhibit superior vitality and growth potential than their parents. This concept has been used as a theoretical basis for crop breeding for over 150 years to enhance agricultural yield. Classical theoretical models, including dominance, overdominance, and epistasis, have been used to explain heterosis [1–5]. However, because heterosis is closely associated with agronomic traits and growth environments, these models do not fully capture the complexity of heterosis [6–8].

Advancements in sequencing technology in the last two decades have shed light on allele-specific expression (ASE), where the imbalance in allele expression between parents in a hybrid offers insights into heterosis [9–13]. The formation of ASE is a complex process regulated by both epigenetic and genetic variations in response to developmental and environmental conditions [14, 15]. A notable instance is genomic imprinting, an epigenetically driven phenomenon where expression depends on the allele’s parental origin in hybrids [16]. The genetic mechanisms of ASE formation involve transcriptional regulation, post-transcriptional regulation, and translational regulation [14]. For instance, the whole-genome analysis of apples revealed that transposable element insertions in the upstream region of genes affect ASE gene (ASEG) transcription [13]. Similarly, in hybrid rice, nonsense-mediated mRNA decay led to biased consistent expression of alleles [17]. Furthermore, studies in rice and potato have identified a negative correlation between ASE and differences in CHG (H = A, C, or T) methylation [12, 18].

Light, as a pivotal environmental cue, not only drives photosynthesis but also acts as a signaling factor guiding plant growth and reproduction. Plants primarily monitor and absorb far-red (700–750 nm), red (600–700 nm), and blue light (400–500 nm) [19–21], which are crucial for carbohydrate transport, photosynthesis, and terpenoid biosynthesis in maize hybrids, respectively [22]. Continuous light irradiation significantly enhances biomass heterosis [22]. However, the genetic and molecular mechanisms underlying the effect of different light spectra on maize hybrid ASEG remain to be elucidated.

In this study, we comprehensively analyzed ASE by using transcriptome data from reciprocal hybrids and their parent strains, B73 and Mo17, under different light treatments. A total of 5,273 ASEGs were identified, which were categorized into three expression types. Gene Ontology (GO) analysis was performed to identify ASEG functions under different light conditions. In addition, we explored promoter variations in heterosis-related ASEGs and assessed how nucleotide diversity contributes to ASEG domestication. These findings not only advance our understanding of maize heterosis but also provide a crucial empirical foundation for crop breeding.

## Methods

### Plant materials

The samples for RNA-seq were maize inbred lines B73 and Mo17 and their reciprocal hybrids B73×Mo17 and Mo17×B73. These experiments were conducted under darkness, far-red, red, and blue light conditions [22]. Briefly, maize seedlings were initially grown in darkness (26 °C) for 6 days, followed by exposure to far-red (737 nm, 2.5 μmol m^− 2^ s^− 1^), red (658 nm, 30.0 μmol m^− 2^ s^− 1^), and blue (447 nm, 6.0 μmol m^− 2^ s^− 1^) light. After 24 h, samples of the seedlings were collected for RNA-seq analysis. Three biological replicates were performed for each sample.

### RNA-seq data processing and ASEG identification

RNA-seq data were obtained from the National Center for Biotechnology Information Sequence Read Archive (SRA) database (https://www.ncbi.nlm.nih.gov/sra; accession no. PRJNA780806). Firstly, raw reads were preprocessed with Trimmomatic software v0.39 [23], where reads with mass less than 30 were filtered out (Fig. S1A). All clean reads from B73 (four treatments, three replications) were mapped to the reference genome of B73 V4 [24] by using Hisat2 v2.1.0 [25] with default parameters. SAMtools software v1.9 [26] was used to remove aligned reads with a mapping quality score of < 30. Single nucleotide polymorphisms (SNPs) were called using the short variant identification pipeline of GATK v4.1.3.0 [27]. In addition, VCFtools software v0.1.13 [28] was employed to filter out variants with an allele frequency of < 0.9 and a depth below 20. The base calls at SNP locations were then substituted with the corresponding bases from the B73 V4 reference genome to reconstruct a pseudo-genome reference through GATK (Fig. S1B).

Clean reads from all samples (four genotypes, four treatments, and three replications) were aligned to the pseudo-genome by using STAR software v2.7.2a [29]. Gene expression levels were quantified as transcripts per million (TPM) (Fig. S1C). GATK and SnpEff software v5.0e [30] were used to identify and annotate SNP variants, respectively. The phASER software v0.9.9.4 [31] was used for ASE identification and annotation at SNP loci, excluding alleles with a depth of < 10. The chi-square test was performed to assess the differential expression of allele abundance, adopting a significance threshold of *P* < 0.05. Gene with imbalanced expression between the parental alleles in F_1_ hybrids (≥ 2 replicates) were designed as ASEG (Fig. S1D). On the basis of their expression patterns, genes were classified into four groups: bias-consistent (BC) ASEGs, bias-specific (BS) ASEGs, bias-reversal (BR) ASEGs, and non-bias genes. The BC ASEGs consistently favored one parental allele under all light conditions. BS ASEGs exhibited a preference for one parental allele under certain light conditions. However, BR ASEGs displayed a directional shift, favoring one parental allele under some light conditions and the other parental allele under other light conditions (Fig. S2).

### Gene expression analysis

Differentially expressed gene (DEG) analysis was performed using the DESeq2 v1.40.1 R package [32], with a false discovery rate (FDR) threshold of < 0.01 and a fold change (FC) of > 1.5. DEGs between hybrids and their parental lines were categorized into three primary groups (additive, complete-incomplete dominant, and overdominant) or further detailed into 12 subcategories (types I to XII) following previously described criteria [22, 33]. Gene expression levels of F_1_ hybrids in additive categories (types I and II) were between those of the two parental inbred lines. Furthermore, gene expression levels of F_1_ hybrids in the complete-incomplete dominant categories (types III, IV, V, and VI) were similar to those in male or female parents. Gene expression levels of F_1_ hybrids in the overdominant categories (types VII, VIII, IX, X, XI, and XII) were lower or higher than those of either parent. In addition, complete-incomplete dominant and overdominant categories were regarded as non-additive genes.

### GO enrichment analysis

GO enrichment analysis of ASEGs was performed using the AgriGO v2.0 database [34], with B73 V4 selected as the background. The enrichment results (*P* < 0.05) were visualized using the clusterProfiler v4.8.2 R package [35].

### Promoter variation analysis

NUCmer v4.0.0beta5 [36] was used to compare between B73 and Mo17 genomes [37]. Filtering results were analyzed using the delta-filter program (parameter ‘-1 -qr’) and parsed using show-coords (parameter ‘-qclT’). Only collinear regions on identical chromosomes were retained. Finally, single-nucleotide polymorphism (SNP) and insertion/deletion (InDel) were identified using show-snps (parameter ‘-ClrT -x 1’). VCFtools was used to extract variants (SNPs, deletions, and insertions) in the 3000 bp promoter region of ASEGs, and frequencies were calculated. Variant information was annotated using SnpEff.

### Nucleotide diversity analysis

Nucleotide diversity (π) was evaluated among 223 accessions, comprising 23 teosinte accessions and 200 maize inbred lines. On the basis of the third-generation Hapmap3 data of *Z. mays* [38], nucleotide diversity was analyzed using the PopGenome v2.7.7 R package [39] with parameters ‘sliding width = 1000’, and ‘sliding jump = 300’. Average nucleotide diversity in the 100-kb upstream region, middle region including ASEGs, and 100-kb downstream region was assessed for each group.

## Results

### Global identification of ASEG expression in maize

**Fig. 1:**
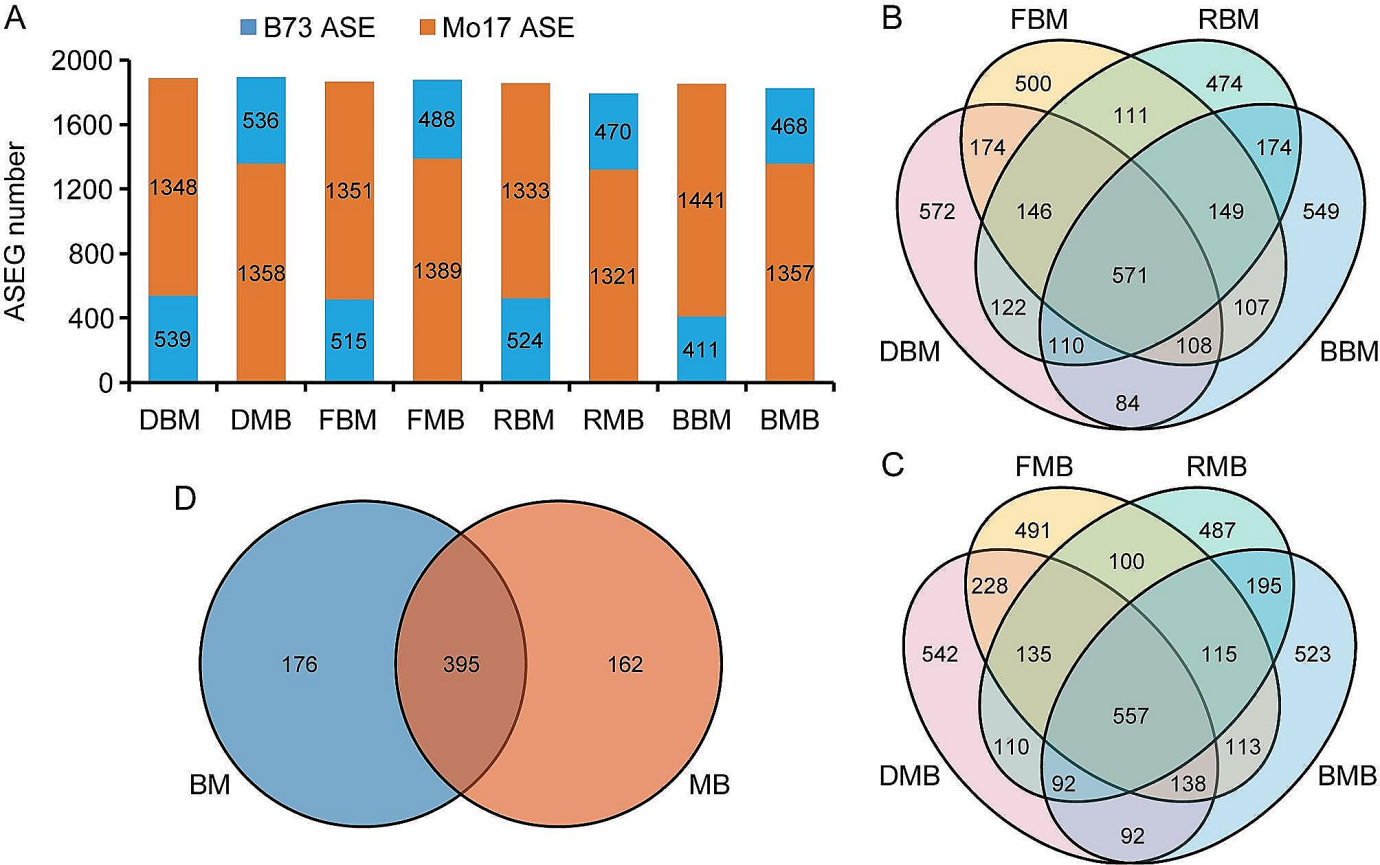
Summary of ASEGs. (**A**) Number of ASEGs in the backcross under various light conditions. (**B**-**C**) Venn diagrams demonstrate the number of ASEGs in BM and MB under various light conditions. (**D**) Overlapping ASEGs in BM and MB under various light conditions. BM and MB represent B73×Mo17 and Mo17×B73, respectively. DBM, FBM, RBM, and BBM represent BM grown in darkness and under far-red, red, and blue light conditions, respectively. DMB, FMB, RMB, and BMB represent MB grown in darkness and under far-red, red, and blue light conditions, respectively

To investigate the effect of light quality on ASEGs in maize hybrids, we examined the transcriptome data of maize inbred lines B73 and Mo17 and their reciprocal hybrids B73×Mo17 (BM) and Mo17×B73 (MB) subjected to darkness or exposure to far-red, red, or blue light [22]. A total of 5,273 ASEGs were identified and categorized into three groups: bias-consistent (BC, 395), bias-specific (BS, 4,754), and bias-reversal (BR, 124) ASEGs (Table S1). No significant difference was observed in the number of ASEGs identified in BM and MB hybrids under various light conditions (Fig. 1A, Table S2). Notably, the number of Mo17-biased ASEGs (1,321–1,441) was significantly higher than those of biased B73 ASEGs (411–539) under all four light conditions (Fig. 1A, Table S2). BM and MB have 571 and 557 common ASEGs under four treatment conditions, respectively (Fig. 1B and C). Among these, 395 BC genes (70 biased toward B73 and 325 biased toward Mo17) were shared between BM and MB (Fig. 1D, Table S3). Despite a strong correlation in bias frequency between the reciprocal hybrids (R^2^ = 0.89) (Fig. S3A), the bias frequency of hybrids exhibited a weak correlation with the gene expression levels of parents (R^2^ = 0.38) (Fig. S3B). These results indicate that hybridization alters gene expression levels from theoretical predictions; however, reciprocal hybrids do not affect the bias of ASEGs.

### Biased B73 and Mo17 BC ASEGs participate in the basal metabolism of hybrids in a functionally complementary manner

**Fig. 2:**
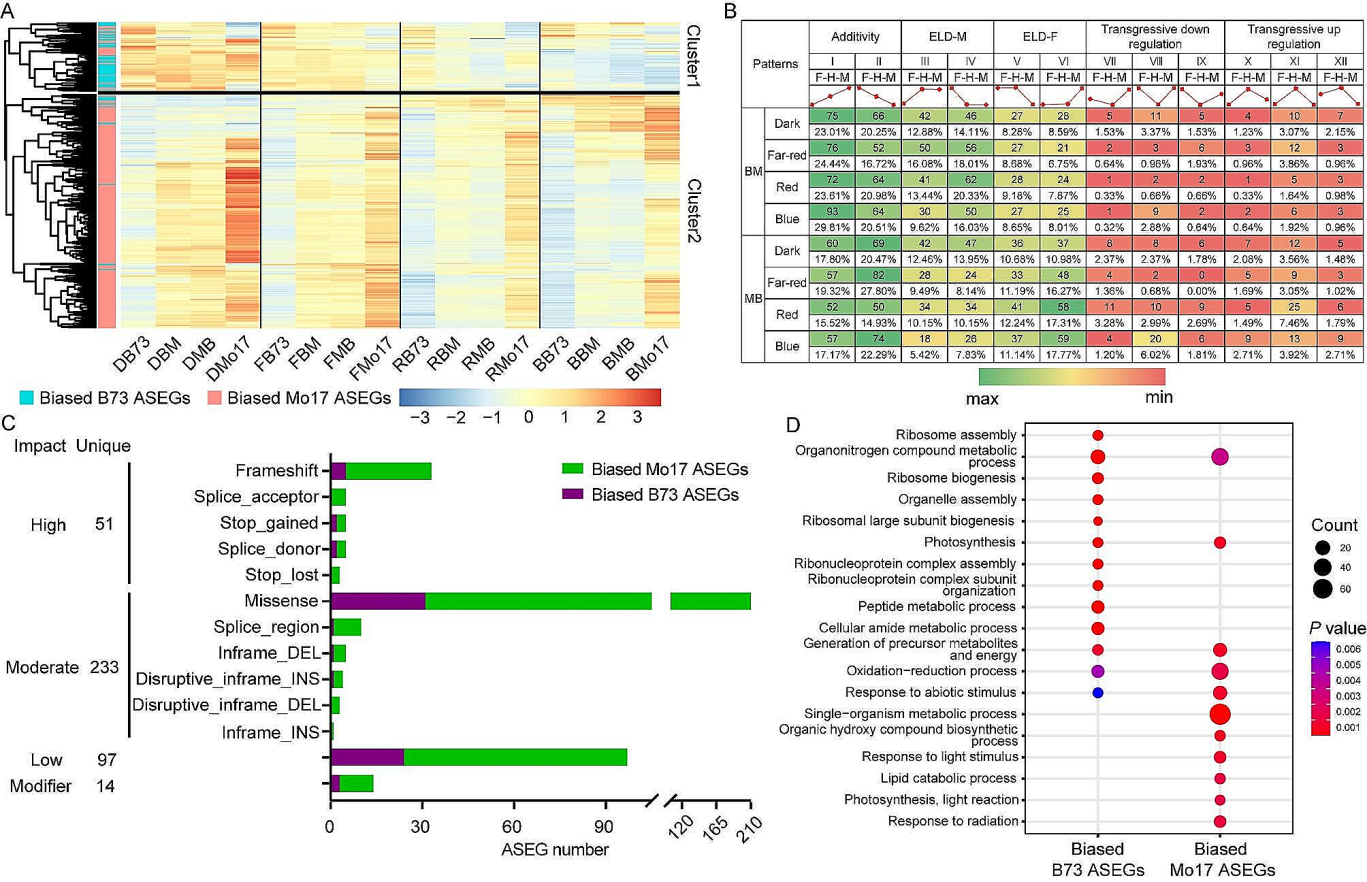
Characteristic analysis of 395 BC genes. (**A**) Expression levels of 395 genes in parents and hybrids. DB73, DMo17, DBM, and DMB represent B73, Mo17, BM, and MB grown in darkness. FB73, FMo17, FBM, and FMB represent B73, Mo17, BM, and MB grown under far-red light condition, respectively; RB73, RMo17, RBM, and RMB represent B73, Mo17, BM, and MB grown under red light condition, respectively; and BB73, BMo17, BBM, and BMB represent B73, Mo17, BM, and MB grown under blue light condition, respectively. (**B**) Expression patterns of 395 genes in hybrids. ELD, expression level dominance; F, female; H, hybrid; M, male. (**C**) Features of BC ASEGs with low, moderate, high, and modifier impact variations. The unique numbers of each impact category are indicated. DEL, deletion; INS, insertion. (**D**) GO enrichment analysis of 395 BC genes

Hierarchical clustering analysis of the 395 BC ASEGs in parents and hybrids revealed their segregation into two main clusters (cluster1 and cluster2). In cluster1, the expression level of the B73 allele was higher than that of the Mo17 allele (90/395). By contrast, in cluster2, the expression level of the Mo17 allele was higher than that of the B73 allele. In addition, the expression levels of alleles in hybrids (305/395) was intermediate between those of the two parents (Fig. 2A). Furthermore, the expression patterns of these 395 BC ASEGs were categorized into 12 subgroups, primarily including additive, dominant (expression level dominance-female, ELD-M; expression level dominance-male, ELD-F) and overdominant (transgressive down/up regulation) effects. Additive effects constituted the majority (30.45–50.32%), followed by ELD-M (13.25–34.08%), ELD-F (15.43–29.55%), and overdominance (4.59–19.70%).

To investigate the potential effects of variations within the BC ASEGs, we compared the coding sequences of the 395 ASEGs between two parents, using B73 as the reference and employing the SnpEff software for annotation. The results showed that 51 (12.91%) of the 395 ASEGs, comprising nine B73 ASEGs and 42 Mo17 ASEGs, contained InDels and SNPs with high impact, potentially leading to protein truncation, dysfunction, or the initiation of nonsense-mediated decay. The most frequent variation was frameshift mutations caused by non-triplet nucleotide insertions/deletions (*n* = 33, 64.71%). Furthermore, 233 (58.99%) of the 395 ASEGs, including 34 B73 ASEGs and 199 Mo17 ASEGs, possessed non-disruptive variants with moderate impacts, which might alter protein function. The predominant category within this group was missense variants (*n* = 210), likely causing changes in protein function but not major structural alterations. The remaining 111 genes comprised low-impact variations (unlikely to alter protein sequences) and modifier-impact mutations (impact undetermined), accounting for 97 (24.56%) and 14 (3.54%) genes, respectively. These mutations do not affect gene expression and structure (Fig. 2C). The diversity of variations suggests that changes in coding sequence are not the primary drivers of ASEGs.

GO enrichment analysis of the 395 BC ASEGs using InterPro classification demonstrated that biased B73 ASEGs were mainly involved in ribosome-related functions, including ribosome assembly, ribosome biogenesis, ribonucleoprotein complex assembly, and ribonucleoprotein complex subunit organization. Biased Mo17 ASEGs were primarily associated with abiotic stress responses and organic compound biosynthesis and metabolism, including photosynthesis, light reaction, organic hydroxy compound biosynthetic process, single-organism metabolic process, lipid catabolic process, and oxidation-reduction process (Fig. 2D). These findings indicate that BC ASEGs originating from different parental sources exhibit functional complementarity in the F_1_ hybrids and participate in basic metabolic processes through additive expression modes. Furthermore, BC ASEGs were consistent in both BM and MB hybrids, suggesting that the complementary manner is the same in reciprocal hybrids.

### BR ASEGs are beneficial for hybrids to adapt to different light environments

**Fig. 3:**
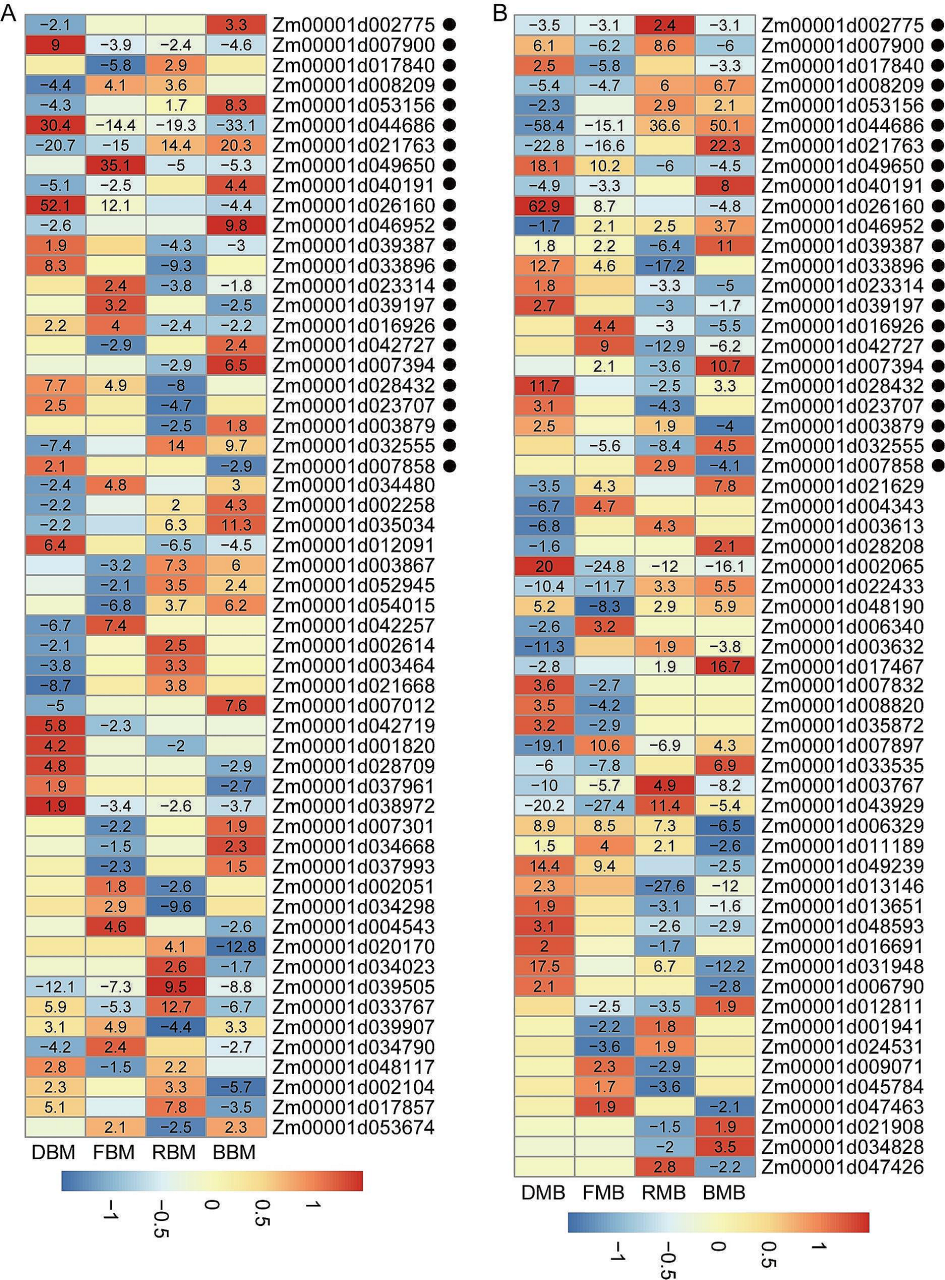
Directions of expression bias for BM (**A**) and MB (**B**) ASEGs under four light conditions. Black dots represent that ASEGs are shared in both BM and MB. The heatmap is labeled using the − log10 (*P* value) of significantly biased SNPs. The marker is a negative number for biased B73 and a positive number for biased Mo17

Under the four light conditions, 124 BR ASEGs were identified. A total of 91 genes exhibited biased transition under different conditions, including 56 ASEGs in BM, 58 ASEGs in MB, and 23 ASEGs common to both reciprocal hybrids (Fig. 3, Table S4). Moreover, 33 genes displayed biased conversion between different genotypes, with genes such as *Zm00001d022421*, *Zm00001d032956*, *Zm00001d034034*, *Zm00001d047349*, *Zm00001d051804*, *Zm00001d042906*, *Zm00001d001966*, and *Zm00001d009717* showing consistent bias in the same genotypes (Fig. S4).

GO functional annotation analysis revealed that the 124 BR ASEGs were predominantly concentrated in processes related to the chloroplast stroma, photosynthetic membrane, and cellular homeostasis (Table S5). Among the ASEGs shared by BM and MB, *Zm00001d044686*, a lipid transport protein, is involved in the mid-stage response to sustained endoplasmic reticulum stress and plays a role in protecting plants from adverse environmental conditions [40]. In BM, the expression level of *Zm00001d044686* showed a Mo17 bias under darkness, whereas it exhibited a B73 bias under the other three light conditions. By contrast, in MB, its expression level was B73-biased under darkness and far-red light but Mo17-biased under red and blue light conditions. *Zm00001d049650* encodes the photosystem II core complex protein PsbY [41]. Its expression in BM and MB was B73-biased under red and far-red light conditions but Mo17-biased under darkness and far-red light conditions. Notably, it showed a Mo17 bias specifically under far-red light conditions. *Zm00001d016926*, homologous to the *Arabidopsis ABC1KB*, is an atypical protein kinase induced by heavy metals [42]. In BM, its expression was Mo17-biased under darkness and far-red light conditions but B73-biased under red and blue light conditions. In MB, its expression was Mo17-biased under far-red light and B73-biased under red and blue light conditions. *Zm00001d023707*, a member of the thioredoxin superfamily, regulates various plant functions, such as germination, development, photosynthesis, and flowering. Its expression in both BM and MB was Mo17-biased in darkness and B73-biased under red light conditions (Fig. 3). These findings suggest that the conversion of allele bias plays a significant role in plant adaptation to diverse light environments.

### BS ASEGs maintain cell stability through both dominant and additive expression

**Fig. 4:**
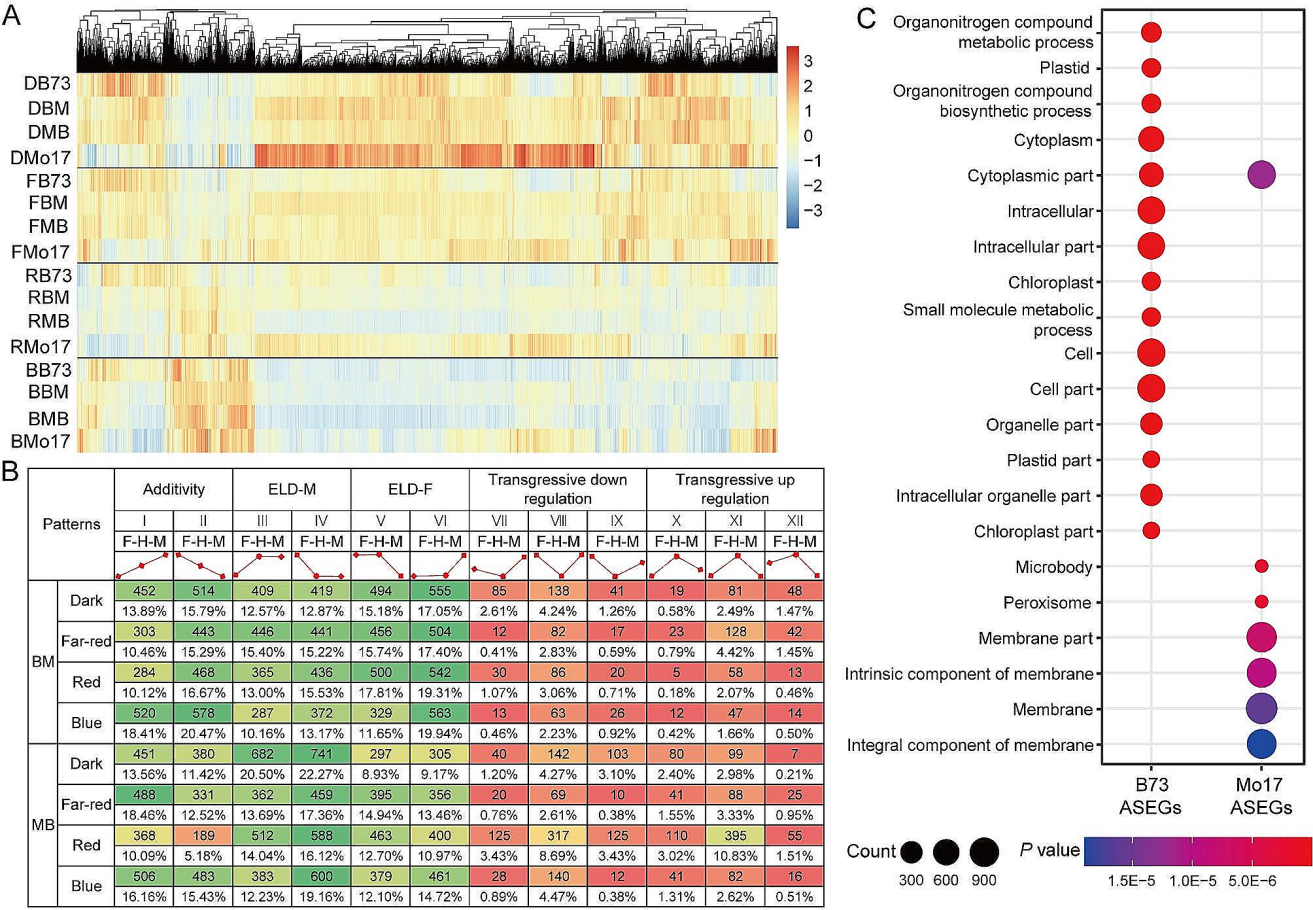
Expression pattern and function analysis of 4,754 BS ASEGs. (**A**) Expression levels of BS ASEGs in hybrids and parents. (**B**) Twelve expression patterns of BS ASEGs in hybrids. ELD, expression level dominance; F, female; H, hybrid; M, male. (**C**) GO enrichment analysis of biased parent-related BS ASEGs

In the BS ASEGs, B73-biased and Mo17-biased genes constituted 28.40% (1,350/4,754) and 71.60% (3,404/4,754), respectively. Notably, the majority of biased genes were exclusive to a single light condition (2,816, 59.23%), with decreasing frequencies under two (998, 20.99%), three (610, 12.83%), and all four light conditions (330, 6.94%) (Fig. S5). Hierarchical clustering analysis indicated that although the expression levels of BS ASEGs significantly varied under different light conditions, the majority of genes in the F_1_ generation mirrored the expression trends of their respective parental lines (Fig. 4A). The 4,754 BS ASEGs were further categorized into 12 types, with gene expression in the F_1_ predominantly characterized by dominant (18.09–42.77%) and additive effects (15.27–38.88%) (Fig. 4B). The proportion of genes exhibiting low parental expression in F_1_ dominant effects (17.05–19.94% in BM and 16.12–22.27% in MB) surpassed those with high parental expression (11.65–17.81% in BM and 12.23–20.50% in MB) (Fig. 4B).

GO enrichment analysis revealed that B73-biased genes were mainly associated with cellular components, such as plastid, cytoplasm, intracellular space, and chloroplast), whereas Mo17-biased genes were predominantly related to membrane structures, including membrane parts, and intrinsic and integral components of the membrane (Fig. 4C). Furthermore, BS ASEG functions showed complete overlap under dark (2,238), far-red (2,220), red (2,166), and blue (2,184) light conditions (Fig. S6). These findings suggest that BS genes contribute to the adaptability of hybrid plants to diverse light environments through dominant and additive expression modes.

### Promoter variations in heterosis-related ASEGs cause ASE formation

**Fig. 5:**
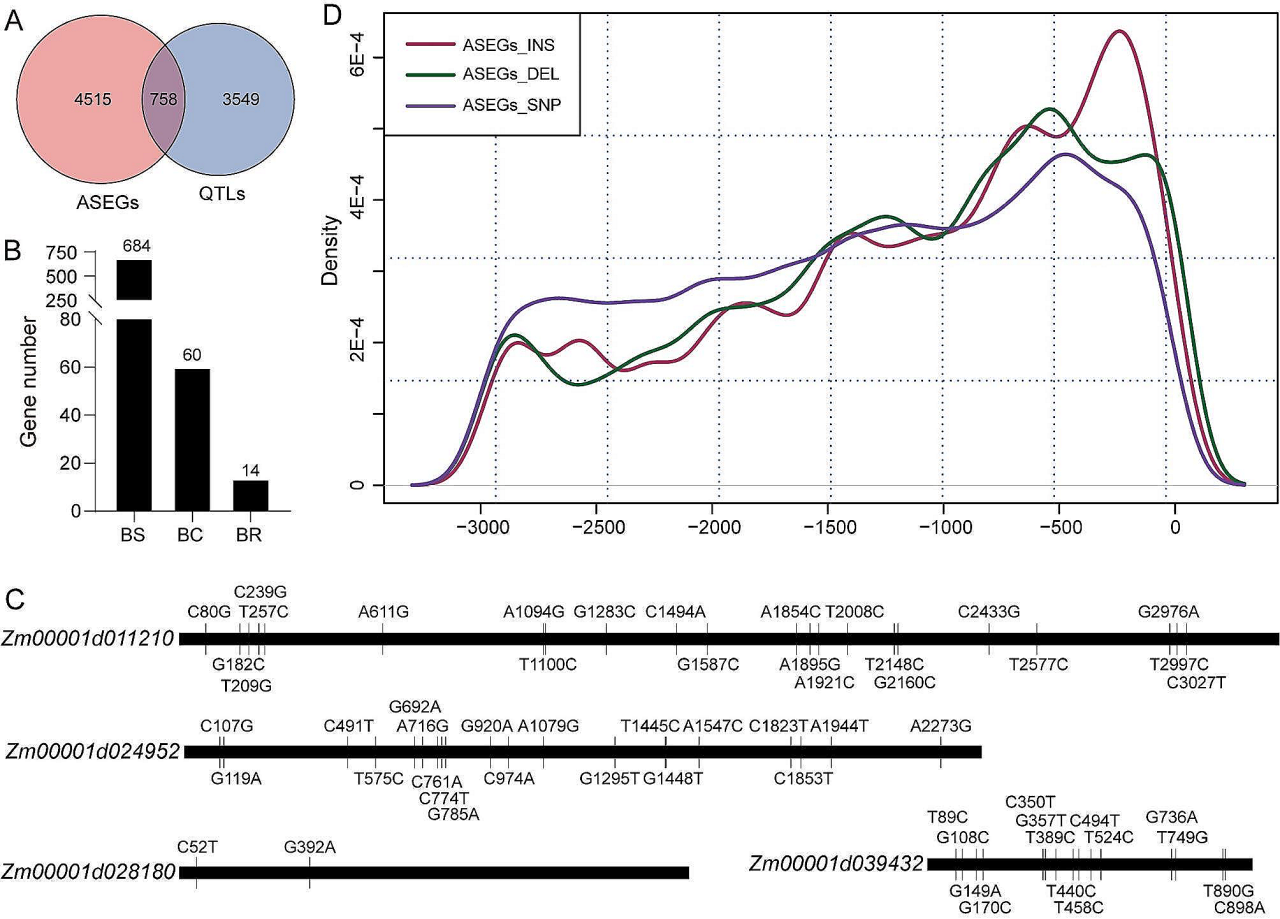
Variations in alleles between parents. (**A**) Venn diagram presenting ASEGs and heterosis-related QTLs. (**B**) Classification of heterosis-related ASEGs. (**C**) Sequence variations in the ASEG coding sequence between B73 and Mo17. B73 served as the reference. (**D**) Differences in the promoter regions of 758 ASEGs between B73 and Mo17. DEL, deletion; INS, insertion

Xiao et al. conducted a genome-wide association analysis on days to tasseling, plant height, and ear weight in 42,840 F_1_ hybrids and identified 4,307 heterosis-related genes [43]. The 758 ASEGs identified in this study were significantly overexpressed in these regions (*P* = 5.04E-3, hypergeometric test) (Fig. 5A), including 684 BS, 60 BC, and 14 BR ASEGs (Fig. 5B, Table S6). When analyzing the coding regions of the 758 heterosis-associated ASEGs, the variation types between B73 and Mo17 sequences were found to be considerably complex. Some genes, such as *Zm00001d011210*, had multiple differential sites (22 loci), whereas others, such as *Zm00001d028180*, had only two SNPs (Fig. 5C). Even in shorter coding regions, significant variations were noted; for instance, *Zm00001d039432* had 15 variant sites. These findings along with previous results (Fig. 2C) suggest that changes in the coding region sequence of heterosis-related ASEGs may not be the primary factor in ASE formation. However, the impact of ASEG coding regions on heterosis cannot be ruled out. To explore whether differences in promoter regions contribute to ASEG formation, 3,000 bp sequences of the promoter regions of the 758 ASEGs between B73 and Mo17 were examined. The results revealed a high frequency of InDels and SNPs in these promoter regions (Fig. 5D), indicating that variations in the promoter region were associated with heterosis-related ASE.

### Ten heterosis-related ASEGs underlie artificial selection during maize domestication

**Fig. 6:**
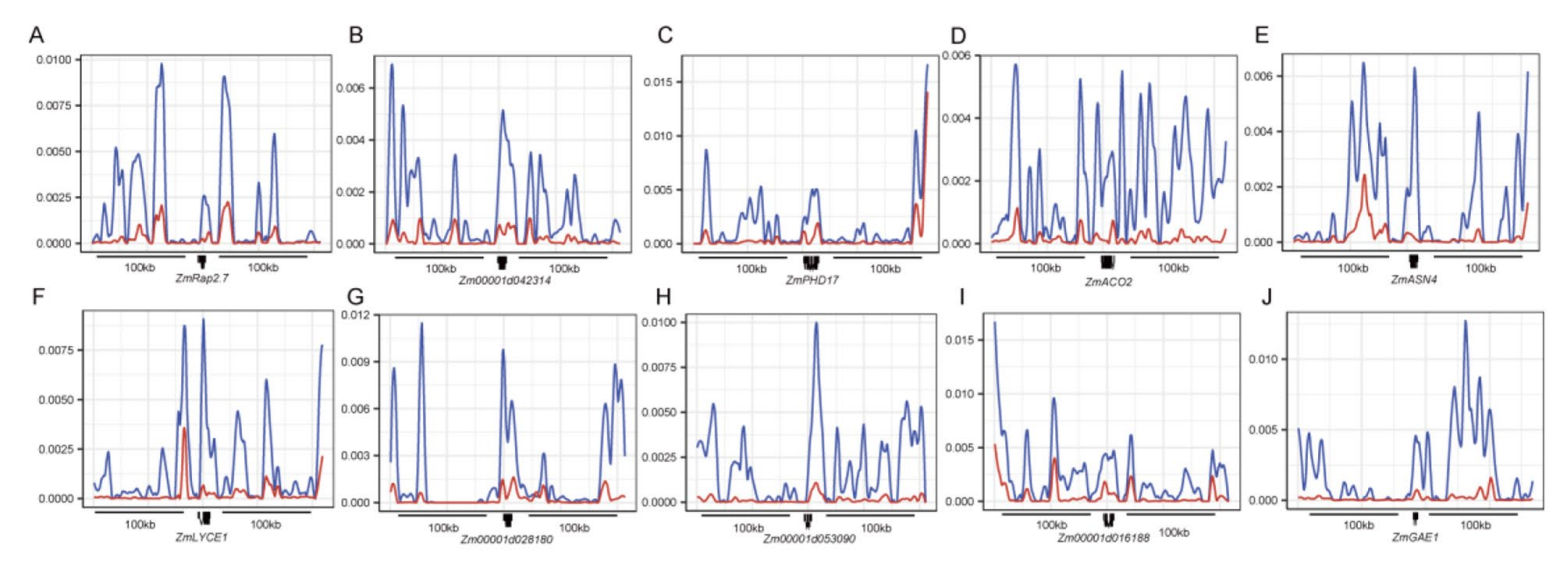
Selective sweep signals of 10 heterosis-related ASEGs. Blue and red lines represent the average nucleotide diversity of heterosis-related ASEGs based on 23 teosinte and 200 maize sequences

Out of 758 heterosis-related ASEGs, 10 have been identified as playing a role in maize heterosis, specifically in stress resistance (*ZmRap2.7*/*Zm00001d010987*, *Zm00001d042314*, and *ZmPHD17/Zm00001d010974*) and yield (*ZmACO2*/*Zm00001d024952*, *ZmASN4*/*Zm00001d047736*, *ZmLYCE1*/*Zm00001d011210*, *Zm00001d028180*, *Zm00001d053090*, *ZmGSK*/*Zm00001d016188*, and *ZmGAE1*/*Zm00001d039432*) heterosis [44]. To determine whether artificial selection has impacted these 10 heterosis-related ASEGs, we examined the nucleotide diversity of 23 teosinte and 200 maize accessions by using hapmap3.2.1 data [38]. For most ASEGs, the average nucleotide diversity in flanking regions was significantly higher in teosinte than in maize accessions (Fig. 6A-J, Table S7). These results suggest that some ASEGs participating in heterosis formation were subjected to artificial selection during maize domestication.

## Discussion

### ASEGs adapt to different light environments through various expression patterns

ASEGs, varying in number and function among different organisms and conditions, play a crucial role in adapting to growth stages [17, 45, 46]. Maize, a typical C4 crop, is sensitive to light. Low red/far-red or low blue light causes the inactivation of phytochromes or cryptochromes, leading to shade avoidance syndrome [47–49]. However, the effects of different light conditions on maize ASEGs is less explored. In this study, the maize transcriptomes of reciprocal crosses (BM and MB) were analyzed under dark, far-red, red, and blue light conditions. The results revealed both the light-general and light-specific expression patterns of ASEGs.

Previous studies have suggested that single additive or dominant mode is the main expression pattern in hybrids [50–52]. In this study, we classified ASEGs on the basis of their expression patterns between parents and hybrids, and determined that BC ASEGs primarily exhibited additive expression (30.45–50.32%). Biased B73 BC ASEGs were mainly involved in ribosome-related functions, whereas biased Mo17 BC ASEGs were associated with abiotic stress responses and organic compound biosynthesis and metabolism. In BS ASEGs (4754/5273, 90.16%), dominant (18.09–42.77%) and additive (15.27–38.88%) expression patterns accounted for a significant proportion. Biased B73 and Mo17 BS ASEGs were primarily associated with cell components and membrane composition, respectively (Fig. 4B and C). Therefore, ASEGs adapt to different light environments through multiple complementary expression patterns, which enhances the robustness of hybrid.

### Promoter variations induce genotype-dependent ASEG formation

ASE differences exist between reciprocal hybrids, a phenomenon observed in rice, maize, and *Arabidopsis* [53–55]. This phenomenon is believed to be caused by genes in the maternal mitochondria and chloroplasts [56]. Other studies have indicated a linear correlation between ASE and genotype during maize seed germination and grain development in reciprocal crosses [57–59]. In our study, reciprocal maize seedlings exhibited a highly genotype-dependent ASE (R^2^ = 0.89) (Fig. 1A, Fig. S3A), indicating its prevalence during the growth and development process of maize hybrids.

Transcription factors regulate gene expression by binding to functional elements in promoter regions. The identification of *ZmBZR1* binding sites through ChIP-seq revealed that motif variants contribute to ASE in F_1_ [60]. Transposon insertions in promoter regions account for 35.4% of *cis*-regulation in maize hybrids [61]. Furthermore, ASE caused by *cis*-regulation significantly differed between high- and low-altitude maize populations [62]. Our comprehensive analysis revealed a high frequency of variations in the promoters of heterosis-related ASEGs, particularly within the upstream 1,000 bp of promoter regions (Fig. 5D). The use of CRISPR-Cas9 editing technology to modify the *CLV3/ESR-RELATED* (*CLE)* promoter changes the expression of network genes, resulting in an increase in maize yield [63]. Thus, understanding the genetic basis of genotype-dependent promoter variations in ASEGs can inform breeding strategies aimed at enhancing maize yield.

### Heterosis-related ASEGs undergo domestication selection

Teosinte possesses abundant genetic diversity and numerous beneficial genes with substantial breeding value. Favorable alleles in teosinte are domesticated into specific alleles in different subgroups through gene infiltration and artificial selection, laying the foundation for heterosis formation [8, 55, 64, 65]. Our analysis demonstrated the significant enrichment of 758 ASEGs in regions associated with heterosis-related genes, with 10 heterosis-related BS ASEGs being significantly domesticated (Fig. 6). Surprisingly, 80 and 229 ASEGs in this study were consistent with heterosis-related genes in buds and seeds [58, 59], respectively. However, no significant enrichment region associated with heterosis-related genes was observed, which could be attributed to the single environmental condition. Future efforts could enrich maize resources and enhance hybrid vigor by analyzing ASEG characteristics and selecting specific alleles.

## Conclusion

This study comprehensively identified ASEGs under different light conditions and analyzed the expression and functional characteristics of three types of ASEGs (BC, BS, and BR). Among them, 395 BC ASEGs maintained basic metabolism in hybrids in a functionally complementary manner. In addition, 124 BR ASEGs contributed to environmental adaptability, whereas 4,757 BS ASEGs ensured cellular stability through both dominant and additive expression. Moreover, 10 ASEGs associated with the heterosis formation have undergone domestication, with variations in promoter regions being one of the reasons for ASEG formation. These findings offer valuable insights for future maize breeding and selection strategies.

### Electronic supplementary material

Below is the link to the electronic supplementary material.

Supplementary Material 1

Supplementary Material 2

## Data Availability

Transcriptome raw data in this study was downloaded from the National Center for Biotechnology Information Sequence Read Archive (SRA) database (https://www.ncbi.nlm.nih.gov/sra; accession no. PRJNA780806).
