# Supplementary material for: Natural variations of heterosis-related allele-specific expression genes in promoter regions lead to allele-specific expression in maize
Source: BMC Genomics. 2024 May 14;25:476. doi: 10.1186/s12864-024-10395-y (PMC11092226; doi:10.1186/s12864-024-10395-y)
Supplement: Supplementary file 1 — Supplementary Material 1 [file 12864_2024_10395_MOESM1_ESM.docx]

# Natural variations of heterosis-related allele-specific expression genes in promoter regions lead to allele-specific expression in maize

Weimin Zhan^1,2^*, Lianhua Cui^1^*, Shuling Yang^1^, Kangni Zhang^1^, Yanpei Zhang^1#^, Jianping Yang^1#^

^1^ College of Agronomy, Henan Agricultural University, Zhengzhou 450002, China

^2^ Guangdong Provincial Key Laboratory of Plant Adaptation and Molecular Design, Guangzhou Key Laboratory of Crop Gene Editing, Innovative Center of Molecular Genetics and Evolution, School of Life Sciences, Guangzhou University, Guangzhou 510006, China

* indicates the authors who contributed equally to this study

^#^ indicates corresponding author

Correspondence: Yanpei Zhang, [zhangyanpei@henau.edu.cn](mailto:zhangyanpei@henau.edu.cn); Jianping Yang, [jpyang@henau.edu.cn](mailto:jpyang@henau.edu.cn)

# Supplementary data

**Additional file 1: Table S1 Summary of ASEGs.**

**Additional file 2: Table S2 Chi-square tests of ASEGs.**

**Additional file 3: Table S3 Summary of BC ASEGs.**

**Additional file 4: Table S4 Summary of BR ASEGs.**

**Additional file 5: Table S5 GO enrichment analysis of BR ASEGs.**

**Additional file 6: Table S6 Summary of heterosis-related ASEGs.**

**Additional file 7: Table S7 Nucleotide diversity of genes.**

**Additional file 8: Fig. S1 Flowchart of ASEG identification process.** (A) Quality control step for filtering raw RNA-seq reads. (B) Construction of the pseudo genome of B73. (C) Identification of SNPs in hybrids. (D) ASEGs are identified through chi-square tests. BM and MB represent B73×Mo17 and Mo17×B73, respectively.

**Additional file 9: Fig. S2 Schematic diagram of ASEG classification.**

**Additional file 10: Fig. S3 Comparison of allele expression between hybrids and their parents.** (A) Proportion of B73 alleles in reciprocal crosses. (B) Proportion of B73 alleles in hybrids and their parents. BM and MB represent B73×Mo17 and Mo17×B73, respectively.

**Additional file 11: Fig. S4 Directions of expression bias for 33 ASEGs between reciprocal hybrids.** The heatmap is labeled with the −log10 (*P* value) of significantly biased SNPs. The marker is a negative number for biased B73 and a positive number for biased Mo17.

**Additional file 12: Fig. S5 Classification of BS ASEGs.** D, B, R, and F represent darkness or blue, red, or far-red light, respectively.

**Additional file 13: Fig. S6 GO enrichment analysis of 4,754 BS genes under different light conditions.**


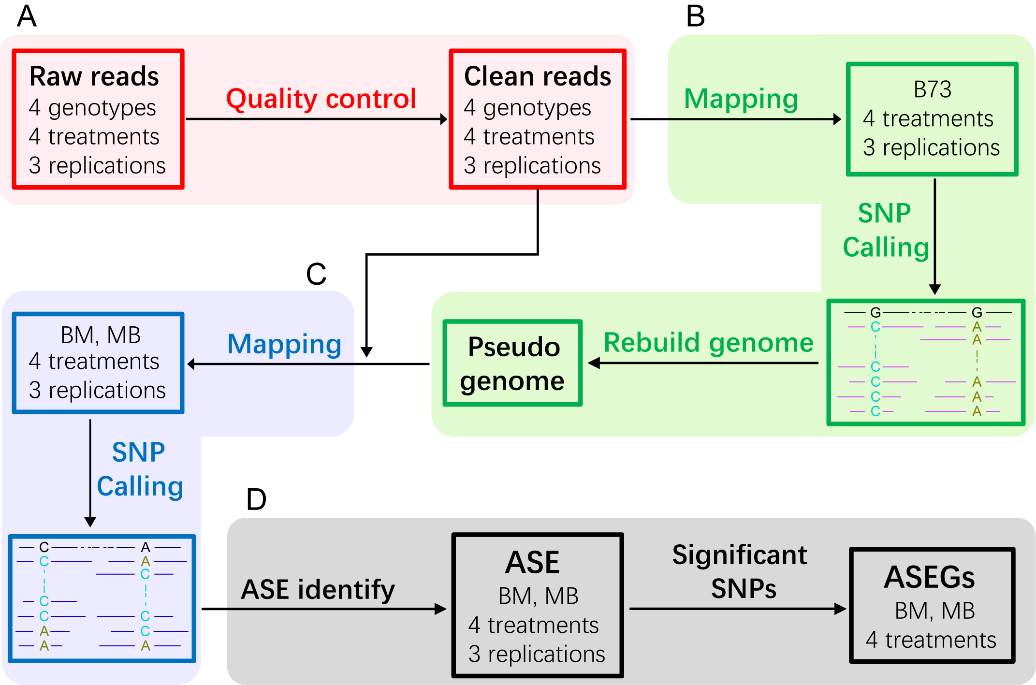


**Fig. S1 Flowchart of ASEG identification process.** (A) Quality control step for filtering raw RNA-seq reads. (B) Construction of the pseudo genome of B73. (C) Identification of SNPs in hybrids. (D) ASEGs are identified through chi-square tests. BM and MB represent B73×Mo17 and Mo17×B73, respectively.


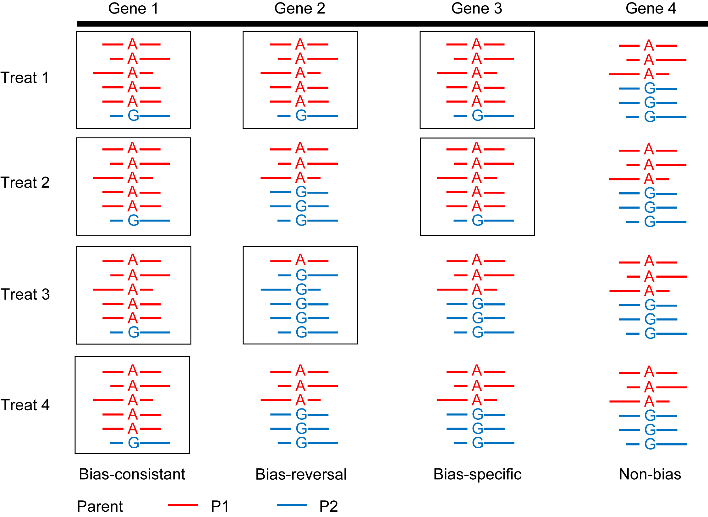


**Fig. S2 Schematic diagram of ASEG classification.**


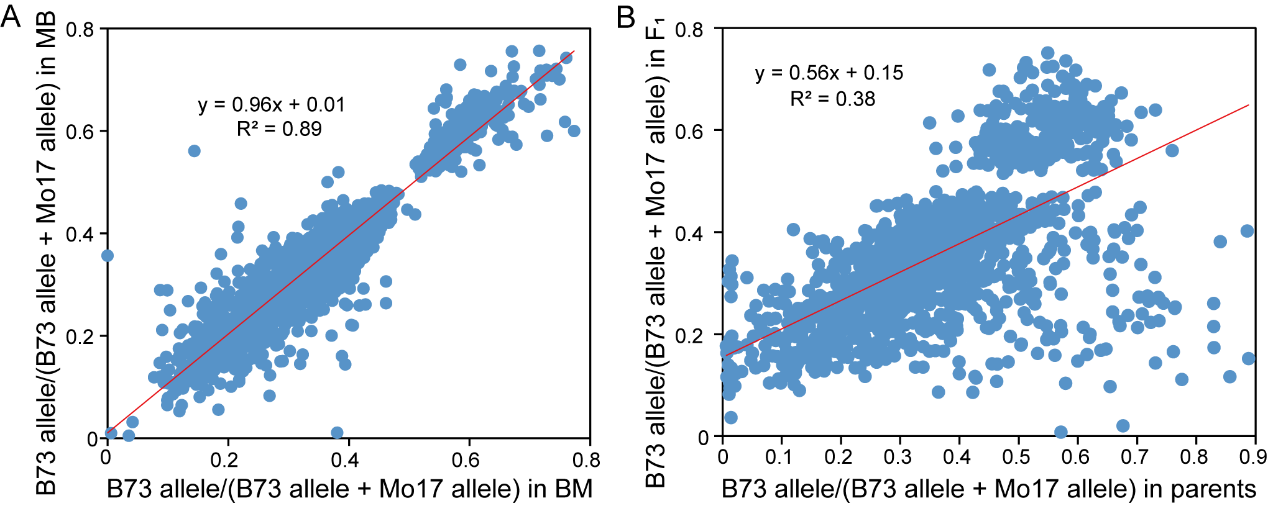


**Fig. S3 Comparison of allele expression between hybrids and their parents.** (A) Proportion of B73 alleles in reciprocal crosses. (B) Proportion of B73 alleles in hybrids and their parents. BM and MB represent B73×Mo17 and Mo17×B73, respectively.


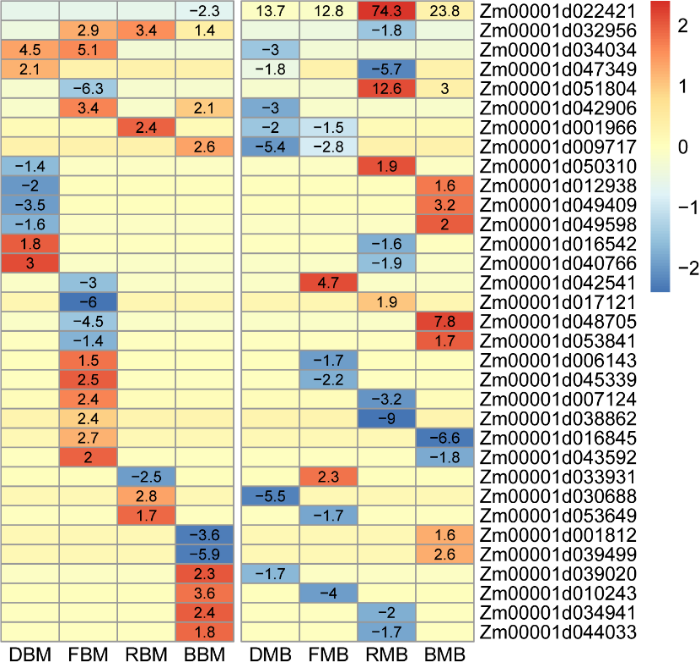


**Fig. S4 Directions of expression bias for 33 ASEGs between reciprocal hybrids.** The heatmap is labeled with the −log10 (*P* value) of significantly biased SNPs. The marker is a negative number for biased B73 and a positive number for biased Mo17.


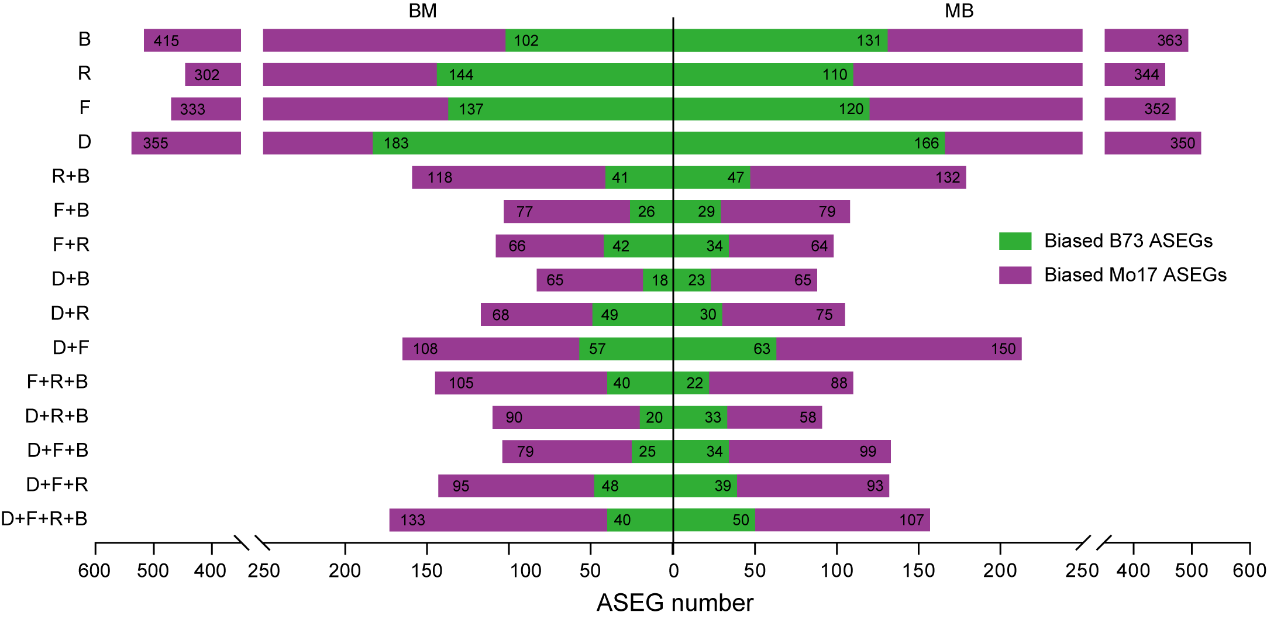


**Fig. S5 Classification of BS ASEGs.** D, B, R, and F represent darkness or blue, red, or far-red light, respectively.


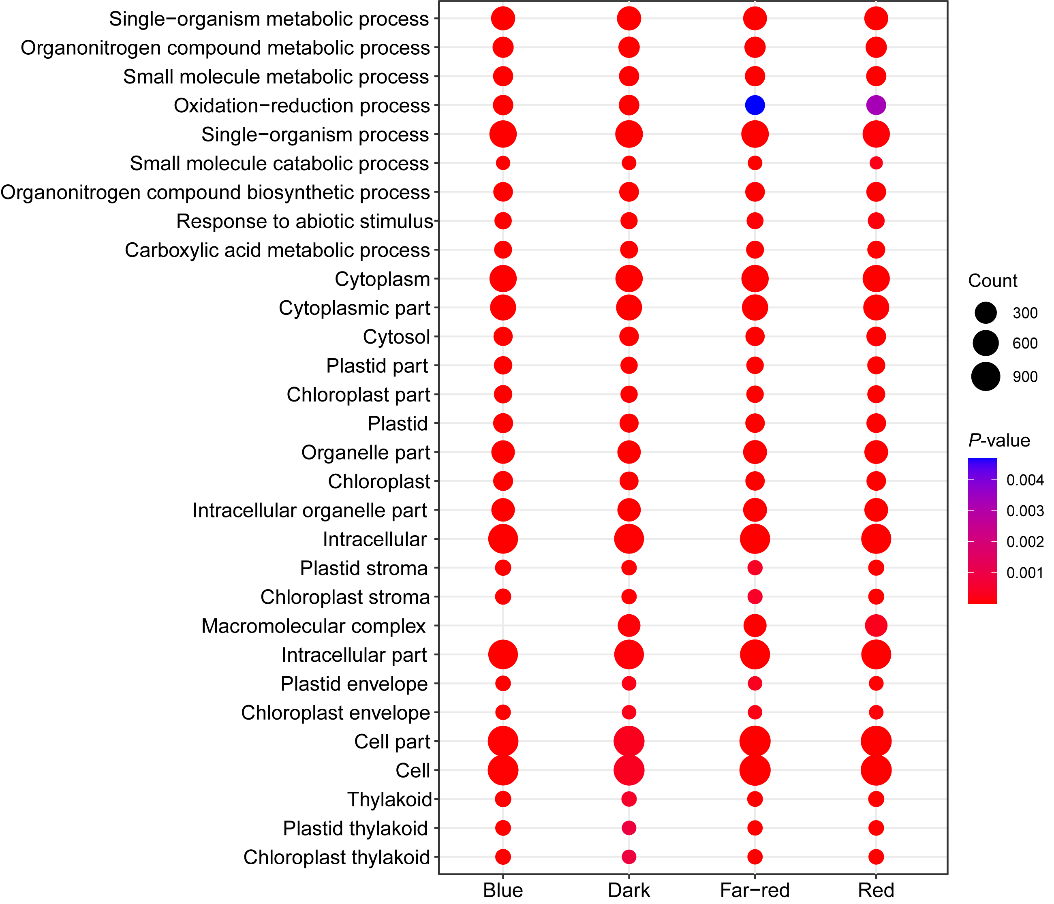


**Fig. S6 GO enrichment analysis of 4,754 BS genes under different light conditions.**
